# Deregulation of the circadian clock constitutes a significant factor in tumorigenesis: a clockwork cancer. Part I: clocks and clocking machinery

**DOI:** 10.1080/13102818.2014.915155

**Published:** 2014-07-14

**Authors:** Kristin Uth, Roger Sleigh

**Affiliations:** ^a^ Centre for Molecular and Cellular Biosensor Research (CMCBR), Abertay University, Dundee, Scotland, UK

**Keywords:** circadian clock, regulation, cell cycle, DNA repair, carcinogenesis

## Abstract

Many physiological processes occur in a rhythmic fashion, consistent with a 24-h cycle. The central timing of the day/night rhythm is set by a master clock, located in the suprachiasmatic nucleus (a tiny region in the hypothalamus), but peripheral clocks exist in different tissues, adjustable by cues other than light (temperature, food, hormone stimulation, etc.), functioning autonomously to the master clock. Presence of unrepaired DNA damage may adjust the circadian clock so that the phase in which checking for damage and DNA repair normally occurs is advanced or extended. The expression of many of the genes coding for proteins functioning in DNA damage-associated response pathways and DNA repair is directly or indirectly regulated by the core clock proteins. Setting up the normal rhythm of the circadian cycle also involves oscillating changes in the chromatin structure, allowing differential activation of various chromatin domains within the 24-h cycle.

## Abbreviations

BER: base excision repair
Cry: cryptochrome
ESC: embryonic stem cells
HMG: high-mobility group
NER: nucleotide excision repair
Per: period
SCN: suprachiasmatic nucleus

## Circadian clocks and clock resetting

Many physiological processes follow a circadian pattern, that is, they occur at specific times in the day/night cycle, and/or their amplitude may vary rhythmically (oscillate) within a cycle close to 24 hours. Such processes include the sleep–wakefulness cycle, the release of hormones, the functioning of the cardiovascular, nervous, digestive and waste disposal systems and others. The core circadian clock machinery exists in every eukaryotic cell (and even some prokaryotes – e.g. Cyanobacteria.[1]), but the overall regulation and coordination is carried out at a central level (master clock – in the suprachiasmatic nucleus (SCN) in the hypothalamus) and several autonomous levels (peripheral clocks – in the heart, the lungs, the gut, and other locations).

The circadian clock is adjustable (entrainable) by exogenous (e.g. light, temperature, food, etc.) or endogenous (e.g. hormone release) cues (triggers). Among external clock-adjusting cues, light (daylight as well as artificial) is prominent, as the master clock in the SCN is predominantly entrainable by light. The signal is picked up by the photoreceptors in the retina and sent to SCN via the retinohypothalamic tract. This means that presence of light must be registered by the visual perception to entrain the master clock in the SCN. Illuminance as low as 3–4-fold above basal may be sufficient for adjustment of the SCN clock.[2] The entrainment of peripheral clocks is typically dependent on stimuli other than light – amongst them being changes in ambient temperature, food availability and noise levels. The circadian clock is self-sustained, that is, in the absence of external cues (e.g. constant darkness), the 24-h circadian rhythm set by the internal clocks are preserved, at least for some time after the entraining cues have been withdrawn (unless some of the major molecules involved in circadian rhythm regulation are missing or altered, in which case the cycle may become shorter or longer). Typically, provision of the missing cue/s adjusts the clock back to the 24-h schedule.

The circadian rhythms are cell-autonomous, that is, they are not functions of cell populations only (e.g. tissues, organs and organisms), but are faithfully maintained at single-cell level for some time after the external cues have been removed.[3] In cells maintained for a long time in *in vitro* culture, however, the circadian rhythms may be lost or reset by exogenous cues (e.g. different temperature, different O_2_ and CO_2_ levels, specific components of the nutrient medium, frequent medium exchange, etc.).[4] Immortalized cell lines may need additional cues (e.g. from the nutrient medium) to maintain their rhythmicity *in vitro*.[4,5]

In an environment deprived of a major entrainment cue (e.g. insufficient light during the day) or environment providing atypical cues (e.g. light at night), circadian rhythms may be seriously disrupted *in vivo*, especially when the disrupting factor(s) are chronically present or when they are frequently but irregularly present. It is known that light pollution may significantly affect the physiology and behaviour of animals – for example, the population density and swimming depth in fish; the post-hatching behaviour of marine turtles; and the mating behaviour in birds.[6–9] In humans, chronic insomnia at night, sleep fragmentation and/or excessive daytime sleepiness commonly occurs in medical personnel doing frequent and/or rotating night shifts and in aircrew members doing frequent long-haul flights crossing several time zones. Sometimes, the consequences of disruption of circadian rhythms may be significantly more severe (e.g. proneness to accidents because of episodes of microsleep during the typical ‘activity phase’ (independent of sleep deprivation); predisposition to multifactorial diseases and conditions; poorer outcomes when the disease has already developed, and others.

## Core machinery of eukaryotic circadian clock

The core machinery of the circadian clock in eukaryotic cells consists of several proteins forming a feedback loop, which is regulated not only at the transcription level, but also by post-translation modifications.[10–12] The exact composition of the set of clock-controlled genes controlled may vary between different tissues, although the total percentage is on the average 5%–10%.[13] The promoters of the clock-regulated genes are usually GC-rich.[14] Core clock proteins activate their target proteins by binding to specific DNA sites in their regulatory regions (usually, E-boxes – with the consensus sequence CAYGTG, where Y = pyrimidine and D-boxes, with consensus sequence RT(G/T)AYGTAAY, where R = purine.[15] Other regulatory elements overrepresented in promoters of clock-controlled genes are sites for binding non-histone proteins (e.g. high-mobility group (HMG) type); the pro-proliferative proteins E2F and c-Myc; STAT1 (member of the Jak-Stat signalling pathway); and others.[14] Many of these proteins also exhibit a circadian pattern of expression and/or are directly regulated by the core clock machinery.

The mammalian proteins Clock, Bmal1 (Arntl1) and Npas2 (the latter is present in the SCN only) are positive regulators of the feedback loop. A heterodimer made of Clock and Bmal1 (or Npas2) binds to and activates a set of light-inducible genes specific to the tissue. The core negative regulators of the circadian feedback loop are the cryptochrome (Cry) proteins (from ‘cryptochrome’) Cry1 and Cry2 and period (Per) proteins (from ‘period’) Per1, Per2 and Per3. These proteins have light-dependent as well as light-independent functions [16] and their expression is activated by the binding of the Clock-Bmal1 heterodimer to E-box sequences in their promoters. Per and Cry proteins form a complex that suppresses their own transcription, as well as the expression of Clock and Bmal1, thus closing the feedback loop.[17,18]

Besides the core circadian clock machinery, there are also other proteins functioning in the regulation of the diurnal rhythm in higher eukaryotes – e.g. Tim1 (Timeless), Dec1 and Dec2, and others.[19,20]

## Clockwork regulation of the cell cycle and the role of persistence of unrepaired DNA damage for adjustment of the circadian clock

Cell division normally follows a circadian pattern in eukaryotic organisms. For example, the rates of proliferation of the epithelial cells in human rectal crypts are higher at night and lower during the day.[21] The differentiation of the epidermal progenitor cells in the upper layers of the skin is dependent on circadian rhythms.[22] The haematopoietic cell niche is also regulated in a circadian fashion with regard to production of blood cells, the targeting of cells at different stages of maturation to different topological and functional locations, and the destruction of aged cells.[23,24] For a while, it was believed that the rhythmic control of cell cycle concerned mainly tissues with rapid turnover. Recently, however, it was reported that even cells that are relatively rarely replaced, such as adult stem cells, may proliferate in rhythmic fashion. In hair follicles from adult mice, the population of stem cells was found to contain discrete stem cell populations that were in the opposite phases of the clock and differentially sensitive to clock-adjusting triggers.[25] The neural precursors in the subgranular zone of adult hypocampus divide slowly but rhythmically, with the entry and exit of the cell cycle controlled by Bmal1 and Per2.[26] The proliferation and the differentiation of mouse oligodendrocyte precursor cells was found to occur in separate timepoints in the activity/rest cycle – during sleep and wakefulness, respectively.[27] The timing of different phases of the cell cycle may vary in different tissues, as it is set by different peripheral clocks. For example, the highest levels of DNA synthesis in bone marrow progenitors in healthy subjects were found to be around noon and in the afternoon [28], whereas cells from human colonic mucosa typically enter the G1 phase of the cell cycle in late afternoon/early evening (around 6 pm) and the S phase later in the night.[29]

The establishment of the circadian rhythm in the developing embryo occurs relatively early in development, but only after the onset of differentiation in the populations of progenitor cells (presumably, because clock-regulated genes may significantly vary in different tissues and the specificities of the diurnal rhythm may be different in different cell types). At present, the proliferation of embryonic stem cells (ESC) maintained in the undifferentiated (pluripotent) state is thought not to be controlled by circadian mechanisms.[30,31] Only after the cell is allowed to differentiate, the typical pattern eventually surfaces. Nevertheless, some of the accessory circadian rhythm proteins (e.g. Tim1) have been found to play a role in developmental apoptosis, facilitating the formation of early embryonic structures such as the proamniotic cavity.[32] Most of the studies of the maintenance of circadian rhythms in cultured cells, however, have been carried out in rodent ESC. The latter may exhibit properties that are very different from the properties of ESC from other mammals and specifically from ESC from primates and humans.[33] It is still unclear whether cells of very early human embryos and cultured human embryonic stem cells (hESC) are actually controlled by circadian rhythms.

The levels of expression of many proteins with roles in carcinogenesis may be dependent (directly and/or indirectly) on the circadian cycle. Some are, for example, products of known proto-oncogenes (e.g. the c-Myc family of proteins); genes coding for proteins directly involved in the transition through the major checkpoints of the cell cycle (checkpoint kinases, cyclins and others); tumour-suppressor genes (e.g. genes coding for CDK inhibitors – *CDKN1A* (coding for the p21 (Waf1) protein and *CDKN2A* (coding for p16 (INK)); genes coding for products functioning in the maintenance of genomic integrity (Ataxia telangiectasia mutated (ATM), p53, HMG of nonhistone proteins, etc.) and genes encoding pro-apoptotic proteins and apoptosis modulators (BCL-2 family – Bax, Puma and Gadd45). Disruption of the circadian rhythm of cell division is considered important component in neoplastic transformation, as DNA damage is a powerful signal for adjustment of the circadian clock. The presence of DNA damage is usually associated with cell cycle arrest for attempted repairs and/or induction of apoptosis (if the damage is assessed as irreparable). Repair activities are usually carried out prior to the S-phase of the cell cycle to avoid transmission of mutations to the progeny by replication of damaged DNA templates. Of course, there are post-replication mechanisms for induction of cell cycle arrest, but the G1/S checkpoint is usually the most stringent cell cycle checkpoint. The S-phase in eukaryotic cells is, as we mentioned above, usually carried out at night. The presence of DNA damage is currently believed to constitute a specific cue for adjustment of the circadian rhythm.[34] Presence of damage in DNA usually brings about an advancement in the phase of the circadian rhythm, as the damage must be repaired before the cell could proceed with replication. The mechanism of DNA damage-associated resetting of the biological clock is conserved in all eukaryotes.[35] It involves virtually all major signalling pathways associated with presence of damage – the ATM/Ataxia telangiectasia and Rad3-related protein (ATR) pathway and the p53-associated pathways for induction of cell cycle arrest and/or apoptosis but may also directly modulate the expression of proteins functioning in cell cycle checkpoints – cyclins, checkpoint kinases; factors stimulating cell proliferation (e.g. c-Myc), and others. 

*Per2* mutant mice display a phenotype of accelerated aging (progressive fur graying) after they have received a non-lethal dose of ionizing radiation (an agent producing double-strand breaks), similar to the Atm heterozygous phenotype.[36–38] This was interpreted as signal suggesting that the functions of Per1 and Per2 proteins and Atm converge to a common pathway.[36,38] Later, it was shown that induced expression of Per1 in cancer cells resulted in phosphorylation of Chk2 even in the absence of DNA damage.[39] Per1 overexpression in human cancer cell lines inhibits tumour growth. In human cell lines transfected with Per1, the proportion of cells in S-phase decreased rapidly, while the proportion of cells in G2/M phase increased.[39] Per1 and Per2 proteins are implicated in the regulation of the ATM-Chk2/ATR-Chk1 DNA damage response pathways.[40] ATR and ATM are serine/threonine kinases that function together as part of the early response system after occurrence of DNA damage, phosphorylating the Rad17 protein.[41–44] ATM is activated predominantly by double-strand breaks whereas ATR may be activated by single-stranded DNA as well (e.g. stalled replication forks). In response to DNA damage, ATM phosphorylates the checkpoint kinase-2 (Chk2), whereas ATR phosphorylates checkpoint kinase-1 (Chk1).[44] Catalytically active Chk2 acts upstream of p53, stabilizing it by phosphorylation and eventually producing cell cycle arrest in G1 in response to DNA damage.[45,46] Chk1-mediated damage-associated pathways typically cause cell cycle arrest in S and G2 phase.[46] The accessory core clock protein Tim1 is essential for ATR-dependent Chk1 activation and S-phase arrest.[47]

ATM/ATR-mediated damage signalling usually results in S-phase advancements, with the magnitude of the advancement dependent on the time of the day when the damage occurred. In mouse cells treated with agents causing double-strand breaks (activating ATM-associated pathways), Per2 was degraded early after the occurrence of DNA damage (early responder) and the next clock phase was advanced, that is, it began earlier than normally expected. The magnitude of the advancement was largest in cases when DNA damage occurred at the time of *de novo* synthesis of Per2 (during the day) and smallest at the peak of Per2 (late in the night).[35] In the same study, UV damage (associated with ATR-mediated response pathway) was shown also to cause phase advancement in mouse cells, with magnitude of the advance dependent on the timing of damage occurrence. 

The tumour-suppressor protein p53 regulates, directly or indirectly, between 2% and 4% of all genes in the mammalian genome.[48,49] p.53 is one of the downstream targets for phosphorylation by ATM in the DNA damage response pathway. p53 phosphorylation by ATM causes its stabilization and accumulation.[43,50,51] ATM also phosphorylates the major negative regulator of p53 (MDM2), decreasing its capacity for exporting p53 from the nucleus.[52,53] p53 may regulate directly the expression of *Per2* via binding to a conserved response element in its promoter.[54] The p53 response element in the *Per2* gene partially overlaps with the E-box element responsible for the transcriptional activation of Per2 expression by the Bmal1/Clock dimer. It was proposed that in the presence of damage, stabilized p53 blocks Bmal1/Clock from binding to the *Per2* promoter, leading to inhibition of the expression of *Per2* and advancement of the subjective day phase.[54] Mouse models lacking p53 have a near-normal circadian rhythm, but they exhibit shortening of the normal 24-h cycle and their biological clock may not be reset correctly by light cues. In cultured cells with p53 knockout, the amplitude of the circadian rhythm is greatly reduced.[55]

Stimulated overexpression of Bmal1 may inhibit tumour growth. The activity of the Bmal1 promoter is strongly enhanced upon DNA damage, but independently of the moment in the subjective day the damage occurred.[35] Bmal1 responds late (several hours) after damage has occurred, activating the ATM-associated damage response pathways.[35,38] Bmal1 stimulates the expression of p53, whereas the Cry proteins downregulate it.[56]

Cry proteins may play a role in the induction of apoptosis by the p53-independent pathway. Mouse cells deficient in both Cry proteins as well as p53 were much more prone to apoptosis after genotoxic challenge than p53-deficient cells that had functional copies of the Cry genes.[55]

Treatment with genotoxic agents usually produces oscillating elevations in the levels of pro-apoptotic proteins. In cells of the same basic type, but with varying levels of differentiation, the amplitude of circadian oscillations of mRNA and protein associated with apoptotic response may be different. A study carried out in mouse haematopoietic cells from bone marrow showed that treatment with ionizing radiation occurring at night resulted in higher p21 and Mdm2 mRNA levels than the levels produced by irradiation occurring during the day.[57] At the same time, no significant differences in the levels of the Bax or Puma were observed in cells from bone marrow irradiated at different times of the day. In cells isolated from peripheral blood, the amplitude of the increase in the levels of p21, Mdm2, Bax and Puma after irradiation occurring during the day were significantly higher than the levels in nighttime irradiation.[57] The level of the p53-regulated cell cycle inhibitor Gadd45α oscillates in a circadian manner, with its expression being directly regulated by Clock.[13,38]

c-Myc is one of the positive regulators of the cell cycle that is directly controlled by the proteins of the clock core complex. c-Myc is a cellular proto-oncogene, a transcription factor, modulating (most commonly – activating, but also repressing) the expression of multiple downstream genes directly implicated in cellular proliferation. The mouse c-Myc gene contains an E-box, where the Clock/Bmal1 or the Clock/Npas2 complex normally bind, inhibiting the transcription of the gene.[35] In Per2-deficient mice, the levels of Bmal1/Npas2 or Bmal1/Clock are reduced, which results in derepression of the expression of c-Myc.[36,38] Following gamma-irradiation, the p53-mediated apoptosis mechanism in Per2-deficient mice does not function adequately [36] and as a result, c-Myc expression is not downregulated and the damaged cells are capable of bypassing the cell cycle checkpoints regardless of the presence of DNA damage, increasing the risk for neoplastic transformation. Deregulation of the expression of c-Myc is believed to constitute a major pathogenetic mechanism in the establishment of the cancer-prone phenotype of Per2-mutant mice, and a risk factor for tumour growth in wildtype mice with disrupted circadian cycle.[38,58] As the mechanisms of induction of DNA repair and apoptosis are, however, partially redundant, elevated levels of c-Myc may route the damaged cells directly to apoptosis, without induction of cell cycle arrest for attempted repair. This may be implemented by c-Myc-mediated suppression of the expression of the p21 (Waf1,Cip1), a CDK inhibitor inducible by stabilized p53.[39] It is believed that *Per1* overexpression inhibits tumour growth by making damaged cells more susceptible to apoptosis via induction of c-Myc/repression of p21.[59] Dec1 and Dec2 proteins repress Clock/Bmal1-induced transactivation of the Per1 promoter in the mouse via direct interaction with Bmal1 as well as by competing with Bmal1 for the E-box elements in the Per1 promoter.[59]

Expression of cyclins also exhibits circadian rhythms. The levels of expression of cyclins D1 and E reach their highest level in early evening (6 pm), in preparation for the G1/S transition; and their lowest level around midnight when the actual cell division is about to start.[29] In the same study, the expression of the cyclin-dependent kinase inhibitors p16 (INK4, or multiple tumour suppressor 1 (MTS1)) and p21 exhibited their lowest levels around noon and at 6 pm, respectively. The accessory circadian factor Dec1 is known to bind directly to cyclin D1 to a designated Dec response element, inhibiting its expression.[20,60] The levels of cyclin B1, and its pairing partner cdc2 (CDK1), chiefly responsible for the transition from G2 to M phase of the cell cycle, and cyclin A2 (functioning in the S and G2 phases) have been found to be deregulated in Cry-deficient mouse models.[61] In Cry-deficient mice with partial hepatectomy, the expression peaks of cyclin B1, cdc2, Bub1 (a serine/threonine kinase essential for the correct chromosome alignment in mitosis), cyclin A2 and the cyclin A2-associated protein p55CDC occurred 8–12 hours later than those in wild-type mice, which resulted in impaired liver regeneration.[61] The expression of Wee1, a negative regulator of the activity of cyclin B/cdc2 complex is directly regulated in a circadian fashion, being activated by the Bmal1/clock heterodimer and inactivated by the Per/Cry complex.[61] Overexpression of Per1 in mouse cells downregulates the expression of cyclin B and cdc2, contributing to the antitumour effect of Per1.[39] The interaction, however, is not direct, rather, it is likely to be p53-mediated, as downregulation was observed only in p53-expressing/Per1 overexpressing cells and not in p53-deficient/Per1 overexpressing cells.[39,61]

Several of the proteins functioning directly in the recognition and repair of DNA damage are expressed in a circadian fashion.[62–64] The levels of the mRNA and protein of the nucleotide excision repair (NER) factor XPA have been found to oscillate within the 24-h cycle, with 5–10-fold changes in amplitude between the lowest and the highest levels; and in anti-phase with the levels of the negative core loop regulator Cry1.[62] The *XPA* gene is believed to be controlled directly by the circadian clock, as its promoter contains E-box elements.[65] In double-mutant Cry1-/Cry2-deficient mice, the levels of Xpa were constitutively high.[65] The levels of other proteins of DNA repair enzymes – specifically, alkylguanine DNA glycosylase (an enzyme of base excision repair (BER)) and O^6^-methylguanine-DNA-methyltransferase (functioning in direct repair) also tend to oscillate within the 24-h cycle, but the amplitude between the highest and the lowest levels in the cycle is significantly lower than the levels seen with Xpa.[66,67] This may be related to the fact that the enzymatic machinery of NER is engaged for repair of virtually all types of lesions, apart from base mismatches and double-strand breaks, whereas enzymes of BER and direct repair usually operate on specific substrates only.

In terminally differentiated cells, the repair of damage occurring in non-transcribed regions of the genome is much less efficient than in transcribed regions.[68] This is presumably associated with the fact that differentiated cells practically never divide; therefore, it is highly unlikely mutations would be transmitted to the cell's progeny.[69] At present, it is unclear whether occurrence of damage in the non-transcribed regions in differentiated cells (where presumably the presence of damage is selectively ignored) would reset the circadian clock as efficiently as damage occurring in the transcribed regions of the genome.

It is now believed that chromatin remodelling is essential for the correct functioning of the circadian clock, as the core feedback loop regulation includes chromatin modifications. The Clock-Bmal1 complex (specifically, Clock) possesses histone acetyltransferase activity, the preferred target for acetylation being the Lys14 residue of nucleosomal histone H3.[70,71] Acetylation of Lys14 has been associated with ‘opening’ of the local chromatin structure.[72] As with most histone acetyltransferases, Clock may also acetylate nonhistone substrate proteins, including its pairing partner Bmal1.[70,73] The acetylation of Bmal1 is carried out on a specific conserved Lys residue (Lys537) and serves as a signal for recruitment of the negative regulator protein Cry1. Bmal1 is acetylated by Clock in a rhythmic manner within the 24-h cycle in the mouse, with the timing of acetylation coinciding with the down-regulation of the transcription of the genes directly controlled by the circadian clock.[70] HMG proteins are a diverse group of non-histone proteins functioning in the supramolecular organization of chromatin and chromatin remodelling.[74,75] HMGB1 functions in virtually all types of DNA repair, facilitating the recognition of the lesion and signalling for recruitment of the DNA repair machinery.[76–78], but may also inhibit DNA repair.[77,79] HMG proteins are normally present at low levels in adult somatic cells.[80–82], but their levels may vary within the 24-h cycle. The levels of Hmgb1 protein oscillates in rat retinal photoreceptor cells, with the expression peaking at the middle of the subjective day and reaching its lowest level at night.[83] Within the nucleus of the photoreceptor cell in the mouse, Hmgb1 co-localizes with acetylated histone H3 (a direct substrate of Clock).[70,83] The circadian changes in the levels of Hmgb1 may therefore reflect the rhythmic changes in chromatin conformation associated with DNA replication and repair. Overexpression of HMGA may suppress DNA repair and directly inhibit the expression of some clock-regulated proteins directly involved in DNA repair, such as XPA.[84,85] The presence of variant alleles of the *Hmga1* gene in mice and humans was shown to be associated with severe early-onset insulin resistance and diabetes type 2.[86,87] The expression of *Hmga1* is up-regulated in the majority of cancers.[78,88–90] It has been proposed that HMG deregulation may increase the risk for neoplastic growth [91,92], but the exact mechanism/s are currently unknown. The role of deregulated circadian rhythm of chromatin remodelling, however, cannot be excluded.

## Conclusions

The mechanisms ensuring the rhythmic oscillation of core clock proteins and clock-regulated proteins are tightly linked and reset by the signalling molecules and pathways involved in DNA damage recognition, damage-associated signalling, DNA repair and apoptosis pathways. The dynamics of the chromatin structure are also regulated in a circadian pattern, allowing for the precise timing of DNA transactions within the 24-h cycle. Deregulation of the normal circadian rhythm (e.g. bedtime misalignment, chronic jet lag, etc.) may increase the risk for development of multifactorial disease (best studied in cancer, but experimental proof exists also for metabolic syndrome/diabetes type 2, cardiovascular and neurological disease); may accelerate cancer progression and/or modify the outcomes for cancer patients.

## References

[cit0001] Johnson CH, Golden SS, Ishiura M, Kondo T (1996). Circadian clocks in prokaryotes. Mol Microbiol..

[cit0002] Kronauer RE, Forger DB, Jewett ME (1999). Quantifying human circadian pacemaker response to brief, extended and repeated light stimuli over the phototopic range. J Biol Rhythms..

[cit0003] Nagoshi E, Saini C, Bauer C, Laroche T, Naef F, Schibler U (2004). Circadian gene expression in individual fibroblasts: cell-autonomous and self-sustained oscillators pass time to daughter cells. Cell.

[cit0004] Kaeffer B, Pardini L (2005). Clock genes of mammalian cells: practical implications in tissue culture. In Vitr Cell Dev Biol Anim..

[cit0005] Farnell YF, Shende VR, Neuendorff N, Allen GC, Earnest DJ (2011). Immortalized cell lines for real-time analysis of circadian pacemaker and peripheral oscillator properties. Eur J Neurosci..

[cit0006] Chepesiuk R (2009). Missing the dark: health effects of light pollution. Environ Health Perspect..

[cit0007] Zheleva M (2012). The dark side of light. Light pollution kills leatherback turtle hatchlings. Biodiscovery..

[cit0008] Bui S, Oppedal F, Korsøen ØJ, Sonny D, Dempster T (2013). Group behavioural responses of Atlantic salmon (Salmo salar L.) to light, infrasound and sound stimuli. PLoS One..

[cit0009] Dominoni D, Quetting M, Partecke J (2013). Artificial light at night advances avian reproductive physiology. Proc Biol Sci..

[cit0010] Lee C, Etchegaray JP, Cagampang FR, Loudon AS, Reppert SM (2001). Posttranslational mechanisms regulate the mammalian circadian clock. Cell..

[cit0011] Sato TK, Yamada RG, Ukai H, Baggs JE, Miraglia LJ, Kobayashi TJ, Welsh DK, Kay SA, Ueda HR, Hogenesch JB (2006). Feedback repression is required for mammalian circadian clock function. Nat Genet..

[cit0012] Kondratov RV, Kondratova AA, Lee C, Gorbacheva VY, Chernov MV, Antoch MP (2006). Post-translational regulation of circadian transcriptional CLOCK(NPAS2)/BMAL1 complex by CRYPTOCHROMES. Cell Cycle..

[cit0013] Oishi K, Miyazaki K, Kadota K, Kikuno R, Nagase T, Atsumi G, Ohkura N, Azama T, Mesaki M, Yukimasa S, Kobayashi H, Iitaka C, Umehara T, Horikoshi M, Kudo T, Shimizu Y, Yano M, Monden M, Machida K, Matsuda J, Horie S, Todo T, Ishida N (2003). Genome-wide expression analysis of mouse liver reveals CLOCK-regulated circadian output genes. J Biol Chem..

[cit0014] Bozek K, Relógio A, Kielbasa SM, Heine M, Dame C, Kramer A, Herzel H (2009). Regulation of clock-controlled genes in mammals. PLoS One..

[cit0015] Gekakis N, Staknis D, Nguyen HB, Davis FC, Wilsbacher LD, King DP, Takahashi JS, Weitz CJ (1998). Role of the CLOCK protein in the mammalian circadian mechanism. Science..

[cit0016] Griffin EA, Staknis D, Weitz CJ (1999). Light-independent role of CRY1 and CRY2 in the mammalian circadian clock. Science..

[cit0017] Langmesser S, Tallone T, Bordon A, Rusconi S, Albrecht U (2008). Interaction of circadian clock proteins PER2 and CRY with BMAL1 and CLOCK. BMC Mol Biol..

[cit0018] Padmanabhan K, Robles MS, Westerling T, Weitz CJ (2012). Feedback regulation of transcriptional termination by the mammalian circadian clock PERIOD complex. Science..

[cit0019] Sangoram AM, Saez L, Antoch MP, Gekakis N, Staknis D, Whiteley A, Fruechte EM, Vitaterna MH, Shimomura K, King DP, Young MW, Weitz CJ, Takahashi JS (1998). Mammalian circadian autoregulatory loop: a timeless ortholog and mPer1 interact and negatively regulate CLOCK-BMAL1-induced transcription. Neuron..

[cit0020] Honma S, Kawamoto T, Takagi Y, Fujimoto K, Sato F, Noshiro M, Kato Y, Honma K (2002). Dec1 and Dec2 are regulators of the mammalian molecular clock. Nature..

[cit0021] Marra G, Anti M, Percesepe A, Armelao F, Ficarelli R, Coco C, Rinelli A, Vecchio FM, D’Arcangelo E (1994). Circadian variations of epithelial cell proliferation in human rectal crypts. Gastroenterology..

[cit0022] Janich P, Toufighi K, Solanas G, Luis NM, Minkwitz S, Serrano L, Lehner B, Benitah SA (2013). Human epidermal stem cell function is regulated by circadian oscillations. Cell Stem Cell..

[cit0023] Casanova-Acebes M, Pitaval C, Weiss LA, Nombela-Arrieta C, Chèvre R, A-González N, Kunisaki Y, Zhang D, van Rooijen N, Silberstein LE, Weber C, Nagasawa T, Frenette PS, Castrillo A, Hidalgo A (2013). Rhythmic modulation of the hematopoietic niche through neutrophil clearance. Cell..

[cit0024] Nguyen KD, Fentress SJ, Qiu Y, Yun K, Cox JS, Chawla A (2013). Circadian gene Bmal1 regulates diurnal oscillations of Ly6C(hi) inflammatory monocytes. Science..

[cit0025] Janich P, Pascual G, Merlos-Suárez A, Batlle E, Ripperger J, Albrecht U, Cheng HY, Obrietan K, Di Croce L, Benitah SA (2011). The circadian molecular clock creates epidermal stem cell heterogeneity. Nature..

[cit0026] Bouchard-Cannon P, Mendoza-Viveros L, Yuen A, Kærn M, Cheng HY (2013). The circadian molecular clock regulates adult hippocampal neurogenesis by controlling the timing of cell-cycle entry and exit. Cell Rep..

[cit0027] Bellesi M, Pfister-Genskow M, Maret S, Keles S, Tononi G, Cirelli C (2013). Effects of sleep and wake on oligodendrocytes and their precursors. J Neurosci..

[cit0028] Smaaland R, Laerum OD, Lote K, Sletvold O, Sothern RB, Bjerknes R (1991). DNA synthesis in human bone marrow is circadian stage dependent. Blood..

[cit0029] Griniatsos J, Michail OP, Theocharis S, Arvelakis A, Papaconstantinou I, Felekouras E, Pikoulis E, Karavokyros I, Bakoyiannis C, Marinos G, Bramis J, Michail PO (2006). Circadian variation in expression of G1 phase cyclins D1 and E and cyclin-dependent kinase inhibitors p16 and p21 in human bowel mucosa. World J Gastroenterol..

[cit0030] Paulose JK, Rucker EB, Cassone VM (2012). Toward the beginning of time: circadian rhythms in metabolism precede rhythms in clock gene expression in mouse embryonic stem cells. PLoS One..

[cit0031] Inada Y, Uchida H, Umemura Y, Nakamura W, Sakai T, Koike N, Yagita K (2014). Cell and tissue-autonomous development of the circadian clock in mouse embryos. FEBS Lett..

[cit0032] O’Reilly LP, Watkins SC, Smithgall TE (2011). An unexpected role for the clock protein timeless in developmental apoptosis. PLoS One..

[cit0033] Arabadjiev B, Petkova R, Momchilova A, Chakarov S, Pankov R (2012). Of mice and men – differential mechanisms of maintaining the undifferentiated state in mESC and hESC. Biodiscovery..

[cit0034] Chen Z, McKnight SL (2007). A conserved DNA damage response pathway responsible for coupling the cell division cycle to the circadian and metabolic cycles. Cell Cycle..

[cit0035] Gamsby JJ, Loros JJ, Dunlap JC (2009). A phylogenetically conserved DNA damage response resets the circadian clock. J Biol Rhythms..

[cit0036] Lee CC (2005). The circadian clock and tumor suppression by mammalian period genes. Methods in Enzymology..

[cit0037] Barlow C, Eckhaus MA, Schäffer AA, Wynshaw-Boris A (1999). Atm haploinsufficiency results in increased sensitivity to sublethal doses of ionizing radiation in mice. Nat Genet..

[cit0038] Fu L, Pelicano H, Liu J, Huang P, Lee C (2002). The circadian gene Period2 plays an important role in tumor suppression and DNA damage response in vivo. Cell..

[cit0039] Gery S, Komatsu N, Baldjyan L, Yu A, Koo D, Koeffler HP (2006). The circadian gene per1 plays an important role in cell growth and DNA damage control in human cancer cells. Mol Cell..

[cit0040] Chen-Goodspeed M, Lee CC (2007). Tumor suppression and circadian function. J Biol Rhythms..

[cit0041] Bao S, Tibbetts RS, Brumbaugh KM, Fang Y, Richardson DA, Ali A, Chen SM, Abraham RT, Wan X-F (2001). ATR/ATM-mediated phosphorylation of human Rad17 is required for genotoxic stress responses. Nature..

[cit0042] Khalil HS, Tummala H, Chakarov S, Zhelev N, Lane DP (2012). Targeting ATM pathway for therapeutic intervention in cancer. Biodiscovery..

[cit0043] Khalil HS, Tummala H, Zhelev N (2012). ATM in focus: A damage sensor and cancer target. Biodiscovery..

[cit0044] Lee CH, Chung JH (2001). The hCds1 (Chk2)-FHA domain is essential for a chain of phosphorylation events on hCds1 that is induced by ionising radiation. J Biol Chem..

[cit0045] Chehab NH, Malikzay A, Appel M, Halazonetis TD (2000). Chk2/hCds1 functions as a DNA damage checkpoint in G-1 by stabilising p53. Genes Dev..

[cit0046] Tang J, Erikson RL, Liu X (2006). Checkpoint kinase 1 (Chk1) is required for mitotic progression through negative regulation of polo-like kinase 1 (Plk1). Proc Natl Acad Sci USA..

[cit0047] Yang X, Wood PA, Hrushesky WJ (2010). Mammalian TIMELESS is required for ATM-dependent CHK2 activation and G2/M checkpoint control. J Biol Chem..

[cit0048] Rahman-Roblick R, Roblick UJ, Hellman U, Conrotto P, Liu T, Becker S, Hirschberg D, Jörnvall H, Auer G, Wiman KG (2007). p53 targets identified by protein expression profiling. Proc Natl Acad Sci USA..

[cit0049] Huart AS, Hupp TR (2013). Evolution of conformational disorder & diversity of the P53 interactome. BioDiscovery.

[cit0050] Banin S, Moyal L, Shieh S, Taya Y, Anderson CW, Chessa L, Smorodinsky NI, Prives C, Reiss Y, Shiloh Y, Ziv Y (1998). Enhanced phosphorylation of p53 by ATM in response to DNA damage. Science..

[cit0051] Chakarov S, Petkova R, Russev GCh (2012). p53 – guardian angel and archangel. Biotechnol Biotechnol Equipment..

[cit0052] Khosravi R, Maya R, Gottlieb T, Oren M, Shiloh Y, Shkedy D (1999). Rapid ATM-dependent phosphorylation of MDM2 precedes p53 accumulation in response to DNA damage. Proc Natl Acad Sci USA..

[cit0053] Loughery J, Meek D (2013). Switching on p53: an essential role for protein phosphorylation?. BioDiscovery.

[cit0054] Miki T, Matsumoto T, Zhao Z, Lee CC (2013). p53 regulates Period2 expression and the circadian clock. Nat Commun..

[cit0055] Ozturk N, Lee JH, Gaddameedhi S, Sancar A (2009). Loss of cryptochrome reduces cancer risk in p53 mutant mice. Proc Natl Acad Sci USA..

[cit0056] Mullenders J, Fabius AW, Madiredjo M, Bernards R, Beijersbergen RL (2009). A large scale shRNA barcode screen identifies the circadian clock component ARNTL as putative regulator of the p53 tumor suppressor pathway. PLoS One..

[cit0057] Ishihara H, Tanaka I, Yakumaru H, Chikamori M, Ishihara F, Tanaka M, Ishiwata A, Kurematsu A, Satoh A, Ueda J, Akashi M (2010). Circadian transitions in radiation dose-dependent augmentation of mRNA levels for DNA damage-induced genes elicited by accurate real-time RT-PCR quantification. J Radiat Res..

[cit0058] Filipski E, Innominato PF, Wu M, Li X M, Iacobelli S, Xian L J, Levi F (2005). Effects of light and food schedules on liver and tumor molecular clocks in mice. J Natl Cancer Inst..

[cit0059] Honma S, Ikeda M, Abe H, Tanahashi Y, Namihira M, Honma K, Nomura M (1998). Circadian oscillation of BMAL1, a partner of a mammalian clock gene Clock, in rat suprachiasmatic nucleus. Biochem Biophys Res Commun..

[cit0060] Bhawal UK, Sato F, Arakawa Y, Fujimoto K, Kawamoto T, Tanimoto K, Ito Y, Sasahira T, Sakurai T, Kobayashi M, Kashima I, Kijima H, Kuniyasu H, Abiko Y, Kato Y, Sato S (2011). Basic helix-loop-helix transcription factor DEC1 negatively regulates cyclin D1. J Pathol..

[cit0061] Matsuo T, Yamaguchi S, Mitsui S, Emi A, Shimoda F, Okamura H (2003). Control mechanism of the circadian clock for timing of cell division in vivo. Science..

[cit0062] Kang TH, Reardon JT, Kemp M, Sancar A (2009). Circadian oscillation of nucleotide excision repair in mammalian brain. Proc Natl Acad Sci USA..

[cit0063] Sancar A, Lindsey-Boltz LA, Kang TH, Reardon JT, Lee JH, Ozturk N (2010). Circadian clock control of the cellular response to DNA damage. FEBS Lett..

[cit0064] Gaddameedhi S, Reardon JT, Ye R, Ozturk N, Sancar A (2012). Effect of circadian clock mutations on DNA damage response in mammalian cells. Cell Cycle..

[cit0065] Kang TH, Lindsey-Boltz LA, Reardon JT, Sancar A (2010). Circadian control of XPA and excision repair of cisplatin-DNA damage by cryptochrome and HERC2 ubiquitin ligase. Proc Natl Acad Sci USA..

[cit0066] Marchenay C, Cellarier E, Lévi F, Rolhion C, Kwiatkowski F, Claustrat B, Madelmont JC, Chollet P (2001). Circadian variation in O6-alkylguanine-DNA alkyltransferase activity in circulating blood mononuclear cells of healthy human subjects. Int J Cancer..

[cit0067] Kim J, Matsunaga N, Koyanagi S, Ohdo S (2009). Clock gene mutation modulates the cellular sensitivity to genotoxic stress through altering the expression of N-methylpurine DNA glycosylase gene. Biochem Pharmacol..

[cit0068] Nouspikel T, Hanawalt PC (2000). Terminally differentiated human neurons repair transcribed genes but display attenuated global DNA repair and modulation of repair gene expression. Mol Cell Biol..

[cit0069] Chakarov S, Russev G (2010). DNA repair and cell differentiation - does getting older means getting wiser as well?. Biotechnol Biotechnol Equipment..

[cit0070] Grimaldi B, Nakahata Y, Sahar S, Kaluzova M, Gauthier D, Pham K, Patel N, Hirayama J, Sassone-Corsi P (2007). Chromatin remodeling and circadian control: master regulator CLOCK is an enzyme. Cold Spring Harbor Symp Quantitative Biol..

[cit0071] Grimaldi B, Nakahata Y, Kaluzova M, Masubuchi S, Sassone-Corsi P (2009). Chromatin remodeling, metabolism and circadian clocks: the interplay of CLOCK and SIRT1. Int J Biochem Cell Biol..

[cit0072] Wang Z, Zang C, Rosenfeld JA, Schones DE, Barski A, Cuddapah S, Cui K, Roh TY, Peng W, Zhang MQ, Zhao K (2008). Combinatorial patterns of histone acetylations and methylations in the human genome. Nat Genet..

[cit0073] Hirayama J, Sahar S, Grimaldi B, Tamaru T, Takamatsu K, Nakahata Y, Sassone-Corsi P (2007). CLOCK-mediated acetylation of BMAL1 controls circadian function. Nature..

[cit0074] Chakarov S, Vassilev P, Tencheva Z, Angelova A (1997). Isolation and properties of human recombinant high mobility group I/Y chromosomal protein. Biotechnol Biotechnol Equipment..

[cit0075] Reeves R, Beckerbauer L (2001). HMGI/Y proteins: flexible regulators of transcription and chromatin structure. Biochim Biophys Acta.

[cit0076] Liu Y, Prasad R, Wilson SH (2010). HMGB1: roles in base excision repair and related function. Biochim Biophys Acta..

[cit0077] Schärer OD (2011). Multistep damage recognition, pathway coordination and connections to transcription, damage signalling, chromatin structure, cancer and ageing: current perspectives on the nucleotide excision repair pathway. DNA Repair (Amst).

[cit0078] Chakarov S, Chakalova L, Tencheva Z, Ganev V, Angelova A (2000). Morphine treatment affects the regulation of high mobility group I-type chromosomal phosphoproteins in C6 glioma cells. Life Sci..

[cit0079] Ugrinova I, Zlateva S, Pashev IG, Pasheva EA (2009). Native HMGB1 protein inhibits repair of cisplatin-damaged nucleosomes in vitro. Int J Biochem Cell Biol..

[cit0080] Chakarov S, Chakalova L, Tencheva Z, Angelova A (1995). HMG I/Y chromosome proteins in developing rat brain cells. C R Acad Bulg Sci.

[cit0081] Zhou X, Benson KF, Ashar HR, Chada K (1995). Mutation responsible for the mouse pygmy phenotype in the developmentally regulated factor HMGI-C. Nature..

[cit0082] Angelova A, Chakarov S, Tencheva Z (1998). Effect of the HMG I/Y chromosomal proteins on the RNA polymerase activity in isolated brain nuclei. C R Acad Bulg Sci..

[cit0083] Hoppe G, Rayborn M (2007). Sears JE. Diurnal rhythm of the chromatin protein Hmgb1 in rat photoreceptors is under circadian regulation. J Comp Neurol..

[cit0084] Adair JE, Kwon Y, Dement GA, Smerdon MJ, Reeves R (2005). Inhibition of nucleotide excision repair by high mobility group protein HMGA1. J Biol Chem..

[cit0085] Adair JE, Maloney SC, Dement GA, Wertzler KJ, Smerdon MJ, Reeves R (2007). High-mobility group A1 proteins inhibit expression of nucleotide excision repair factor xeroderma pigmentosum group A. Cancer Res..

[cit0086] Foti D, Chiefari E, Fedele M, Iuliano R, Brunetti L, Paonessa F, Manfioletti G, Barbetti F, Brunetti A, Croce CM, Fusco A, Brunetti A (2005). Lack of the architectural factor HMGA1 causes insulin resistance and diabetes in humans and mice. Nat Med..

[cit0087] Chiefari E, Tanyolaç S, Paonessa F, Pullinger CR, Capula C, Iiritano S, Mazza T, Forlin M, Fusco A, Durlach V, Durlach A, Malloy MJ, Kane JP, Heiner SW, Filocamo M, Foti DP, Goldfine ID, Brunetti A (2011). Functional variants of the HMGA1 gene and type 2 diabetes mellitus. JAMA..

[cit0088] Chiappetta G, Manfioletti G, Pentimalli F, Abe N, Di Bonito M, Vento MT, Giuliano A, Fedele M, Viglietto G, Santoro M, Watanabe T, Giancotti V, Fusco A (2001). High mobility group HMGI(Y) protein expression in human colorectal hyperplastic and neoplastic diseases. Int J Cancer.

[cit0089] Sgarra R, Rustighi A, Tessari MA, Di Bernardo J, Altamura S, Fusco A, Manfioletti G, Giancotti V (2004). Nuclear phosphoproteins HMGA and their relationship with chromatin structure and cancer. FEBS Lett..

[cit0090] Kostova N, Zlateva S, Ugrinova I, Pasheva E (2010). The expression of HMGB1 protein and its receptor RAGE in human malignant tumors. Mol Cell Biochem..

[cit0091] Erol A (2010). Metabolic syndrome is a real disease and premalignant state induced by oncogenic stresses to block malignant transformation. Med Hypotheses..

[cit0092] Petkova R, Tummala H, Zhelev N (2011). Nothing in excess – lessons learned from the expression of high-mobility group proteins type A in non-cancer and cancer cells. Biotechnol Biotechnol Equipment..

